# Preliminary study of an intestinal bio-robot system based on nerve stimulation

**DOI:** 10.1186/1743-0003-9-68

**Published:** 2012-09-29

**Authors:** Lan Zhu, Hongying Liu, Zhenyu Wang, Xitian Pi, Shengshan Zhou

**Affiliations:** 1 Key Laboratory of Biorheological Science and Technology of Ministry of Education, College of Bioengineering of Chongqing University, Chongqing, 400044, PR China; 2 Key Laboratories for National Defense Science and Technology of Innovative Micro/Nano Devices and System Technology, Chongqing University, Chongqing, 400044, PR China

**Keywords:** Intestinal bio-robot system, Intestinal examination, Nerve stimulation, Locomotion control

## Abstract

**Background:**

Wireless capsule endoscopes for diagnosis and treatment in the gastrointestinal tract face the common problem of active actuation. To tackle this difficulty, a non-invasive intestinal bio-robot system with active actuation based on nerve stimulation was developed.

**Methods:**

This intestinal inspection system utilized a natural organism—the mud eel—to serve as the locomotion mechanism, and it was controlled by a LabVIEW-programmed pulse generator. The exterior control unit was able to actively drive and remotely control the navigation and site-specific anchoring of the organism.

**Results:**

Through in vitro stimulation experiments, a method of controlling the organism’s forward motion was obtained: when the organism was stimulated at the tail, it moved forward at a relatively fast speed and with high repeatability. The stimulator parameters were as follows: amplitude 1.85 μA, frequency 2 Hz, pulse duration 500 μs.

**Conclusions:**

Since this is a preliminary study, considerable work remains to be done. However, the results could provide a solid theoretical basis for further research toward producing a practical intestinal bio-robot for the diagnosis and treatment of the gastrointestinal tract.

## Background

In the non-invasive diagnosis and treatment of the gastrointestinal (GI) tract, wireless capsule endoscopes and intestinal tract robots have attracted considerable attention among researchers in recent years. In 2000, Iddan et al. (Israel) developed a wireless capsule endoscope to capture images of the intestinal tract [1]. In 2001, the Given Imaging Company in Israel produced the capsule endoscope M2A for such image acquisition [2]. Subsequently, the RF System Lab Company of Japan launched a smart capsule that combined an endoscope with the ability to extract alimentary tract fluid and make a site-specific drug release. In 2006, the US SmartPill Corporation developed the pH.p Capsule, which is able to detect pressure, pH value, and temperature within the entire GI tract [3]. However, the movement of most smart capsules depends on natural GI peristalsis, i.e., the movement is passive. Therefore, these capsules lack the ability to control movement and posture.

To overcome such shortcomings, active intestinal robots, which are able to execute active movements and achieve site-specific anchoring within an organism, have been developed. In 1988, Ikuta et al. (Japan) developed a snake-like robotic endoscope that was based on a shape memory alloy servo actuator system [4]. In 1995, Slatkin et al. designed an inchworm-like endoscope based on a pneumatic actuator to collect GI images [5]. In 2007, Yan et al. (China) devised a micro peristaltic robot actuated by a direct current motor [6]; the same year, the same group developed a mini-robot for endoscopy based on a wireless power transfer [7]. Although the drive modes of intestinal robots vary, the active actuation still remains a difficult area, especially the power-supply problem [8]. Worldwide, there has been no good solution into how to develop an intestinal robot with active actuation which could also reach the deep part of the intestinal tract and fulfill site-specific anchoring but without power supply wires.

In light of the above situation, we have developed a novel intestinal bio-robot system based on neural microelectrode control. Research into bio-robots is an interdisciplinary area that includes neurology, informatics, and robotics, and it has emerged as a new attractive direction for robotics as a whole. Studies of bio-robots have been successfully carried out on, among other animals, cockroaches, rats, apes, and even sharks [9]. Our research has involved successful preliminary in vitro stimulation experiments using the mud eel which could provide important reference information for further research.

## Methods

The experimental research on animals followed internationally recognized guidelines.

### System and principle

**Figure 1:**
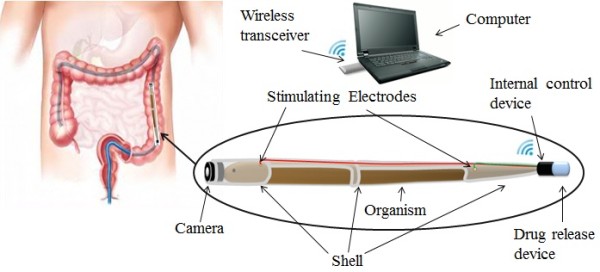
Schematic diagram of the intestinal bio-robot system.

The designed bio-robot system (Figure 1) consisted of two parts: the exterior control unit; and the diagnosis and treatment (D&T) part, which enters the GI tract. The D&T part comprised a camera, a shell, the experimental organism, stimulating electrodes, an internal control device, and a drug-release device. Our chosen organism was mud eel which served as the actuation device of the D&T part, could be remotely controlled by the exterior control unit to initiate actuation and site-specific anchoring.

The shell, which was made of medical silica gel and assembled with stimulating electrodes, consisted of a camera at the front end and an internal control device and drug-release device at the tail end. To keep the organism’s skin wet and reduce the effect on locomotion, the shell was not sealed off (Figure 1). Images taken by the camera were transmitted to the computer by the wireless transceiver. The computer then sent the stimulating signals to the internal control device also via the wireless transceiver. The electrical stimulation was transmitted to the organism by means of stimulating electrodes; in this way, its forward movement could be controlled, and it could be made to stop when it reached the target area of the intestinal tract and release a site-specific drug. Once the task is completed, the D&T part could be withdrawn by stimulating the organism in the same fashion as above.

The organism chosen for the bio-robot system was a mud eel, which belongs to Heterenchelyidae. Mud eels are slender, cylinder-shaped fish without scales and fins; they are very smooth and thus are perfectly shaped to move in long, narrow environments. In addition, they show strong adaptability and are able to survive in water with scarce oxygen for long periods. Studies have shown that when a mud eel was sealed in a bag filled with a certain mass ratio of water and oxygen (mud eel:water:oxygen=1:1:3), it could survive for 24 h [10]. Further, the animals move slowly, are not aggressive, and always avoid external stimulation, which is an advantage in controlling them to move forward, backward, and stop in the intestinal tract.

### Motion control mechanism

The nervous system of mud eels comprises a central nervous system, peripheral nervous system, and vegetative nervous system. The central nervous system consists of the spinal cord and cerebrum; the peripheral nervous system embraces the spinal nerves and cranial nerves; and the vegetative nervous system is made up the sympathetic and parasympathetic nervous systems [11]. A reflex arc includes the receptor, sensory neuron, nerve center, motor neuron, and effector [12].

The exteroceptors of mud eels—lateral lines—are very sensitive. The lateral lines are dominated by the literal nervous system, which is divided into anterolateral line and posterolateral line nerves (PLLNs). The former are a branch of the facial nerves, which dominate the mechanoreceptors of the head; the latter are a branch of the vagus nerves, which dominate the mechanoreceptors of the trunk and tail. The two PLLNs are slightly concave on either side of the trunk [13]. The facial and vagus nerves belong to the cranial nerves, which can transmit impulses to the nerve center [14].

The movement of mud eels depends on muscle contraction. First, each cross section of the spine bends to one side by means of muscular contraction. Then, each cross section bends to the other side, thereby creating a rhythmic alternation that produces the movement. Another form of movement is achieved through the coordinated contraction of adjacent cross sections to form an orderly wave [15].

**Figure 2:**
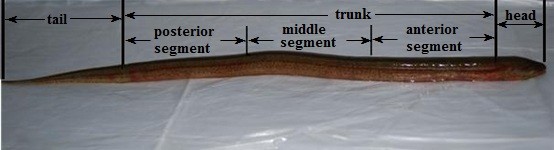
Division of a mud eel's body.

We employed the most frequently used stimulation mode—electrical stimulation—to the anterior, middle, and posterior segments of the PLLN area. Figure 2 shows the division of a mud eel’s body. Excitation produced by electrical stimulation is transmitted to the nerve center in the form of impulses through the vagus nerves. The nerve center analyzes impulses and generates excitation, which is transmitted to motor neurons via the spinal nerves. Then, the excitation is transmitted to muscle cells, which affect the muscle contractions or extensions for the mud eel’s movement.

### Stimulation device

#### Stimulating electrodes

The electrodes utilized here were of two types—wearable stimulating electrodes and implantable stimulating electrodes. The wearable ones were fixed to the surface of the mud eel’s body without invasion and allowed the animal to move without restriction, though we were unable to fix them onto the narrow area of the tail. The implantable electrodes are invasive, yet the minimal invasion does little apparent harm to the organism. Implantable stimulating electrodes could be successfully fixed to the tail, and they also exerted a better stimulatory effect. Additionally, since the implantable electrodes were lighter and did not lead to protrusions on the surface of the organism, they incurred less load on the animal than the wearable ones.

**Figure 3:**
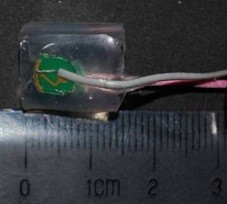
Photo of the surface-mount microelectrode patch.

**Figure 4:**
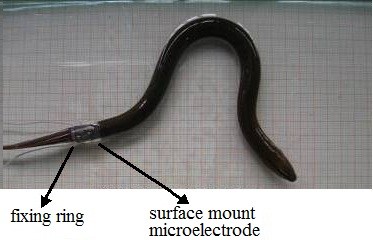
Installation of wearable stimulating electrodes.

The wearable stimulating electrodes utilized here consisted of surface-mount microelectrode patches and a fixing ring. The gold-plated microelectrodes (diameter, 2 mm) were attached with wires and embedded on the surface of the medical silica gel ring (diameter, 10 mm; thickness, 0.5 mm; width, 15 mm) to form a surface-mount microelectrode patch. Since the chosen mud eels were 380±20 mm in length and had a trunk circumference of 38±3 mm, the fixing ring had the following dimensions: diameter, 6 mm; thickness, 0.5 mm; width, 0.5 mm. Figure 3 shows the surface-mount microelectrode patch, and Figure 4 the installation of wearable stimulating electrodes.

**Figure 5:**
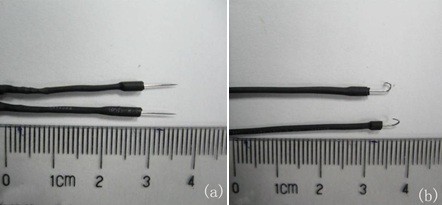
Photo of implantable stimulating electrodes.

The implantable stimulating electrodes consisted of needle-like neural microelectrodes, silicone tubes, and pyrocondensation pipes. The electrodes (diameter, 0.3 mm; length, 35 mm) were made of stainless steel needles. The end of the needle was connected to a copper wire (diameter, 0.3 mm). Two segments of the silicone tubes covered the joint of the copper wire and needle, which was 5 mm in length from the needle tip. The pyrocondensation pipe was used to cover the two segments of the silicone tubes. Figure 5 shows the implantable stimulating electrodes.

#### Stimulator

The stimulator used for the preliminary experiments employed LabVIEW software and the analog output module NI9265 (both National Instruments Corporation, Austin, Texas, USA). We conducted experiments on different body parts of many mud eels to measure the impedance between two stimulating points. In this way, we determined the pulse current for the in vitro stimulating control experiment, which was as follows: amplitude, 1.85 μA; frequency, 2 Hz; pulse duration, 500 μs [12,16].

## Results and discussion

To explore the method of controlling the mud eels’ forward movement in the intestinal tract, the animals were divided into three groups in the in vitro stimulating control experiment. Different stimulating points were tested, and experiments on every stimulating point of each mud eel were repeated three times. The three groups were stimulated under the following conditions: Group 1, in a flexible plastic film tube; Group 2, in a simulated intestinal tract; Group 3, in an in vitro intestine.

### Stimulation experiments in the flexible plastic film tube

Since the intestinal tract is flexible, we used a flexible plastic film tube with a diameter of 60 mm to simulate the intestine. The 12 mud eels in Group 1 were divided into four subgroups with three animals in each.

We put 350 mg fish diazepam into four buckets, each filled with 1 L clear water. After the fish diazepam had dissolved, three mud eels were placed into each bucket. After 5–10 min, the mud eels were anesthetized. They were then removed and assembled with stimulating electrodes in the anterior segment (AS), middle segment (MS), posterior segment (PS) of the trunk, and tail. Subsequently, the water in the buckets was changed to clear water, and the eels were returned to the buckets. After 8–15 min, they were awake and were then placed in the flexible plastic film tube for the experiments. Each stimulation lasted 30 s, and it was repeated after the eel had been returned to the bucket for 5 min.

Double-point stimulation experiments were conducted the day after the single-point experiments. While in anesthesia, the mud eels were assembled with stimulating electrodes as follows: AS and MS; AS and PS; AS and the tail; and MS and PS. The experiments were then carried out in the manner described above.

**Figure 6:**
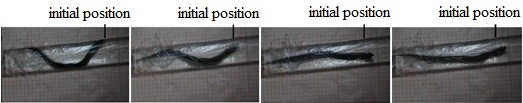
Movement of a mud eel when stimulating the AS of the trunk.

**Figure 7:**
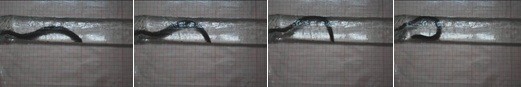
Movement of a mud eel when stimulating the PS of the trunk.

During the single-point experiments, we observed the following motion patterns among the mud eels. (a) When stimulated in the AS, the eels moved forward with a curling motion; thus, the forward displacement was relatively small. Figure 6 shows the movement of a mud eel when stimulating the AS. (b) When stimulated in the MS, the eels also moved forward with relatively small displacement. (c) However, when stimulated in the PS, the eels immediately turned their heads toward the tail, and the entire body assumed an O-shape with no forward movement. Figure 7 shows the movement of a mud eel when stimulating the PS. (d) When stimulated in the tail, the eels twisted and moved forward so fast that the displacement was about 600 mm during the 30-s stimulation.

**Figure 8:**
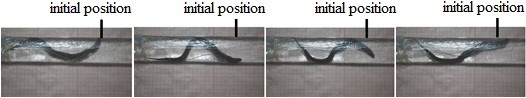
Movement of a mud eel when stimulating the AS and MS of the trunk.

**Figure 9:**
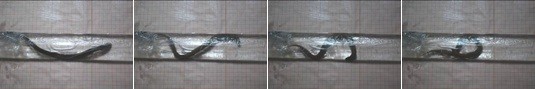
Movement of a mud eel when stimulating the AS and PS of the trunk.

During the double-point experiments, we observed the following motion patterns among the mud eels. (a) When simultaneously stimulated in the AS and MS, the eels clearly moved forward with a displacement of around 110 mm during the 30-s stimulation (Figure 8). (b) When stimulated in the AS and PS, the eels assumed an O-shape with no forward movement (Figure 9). (c) When stimulated in the AS and tail, the eels twisted and curled with little forward displacement. (d) When stimulated in the MS and PS, the eels maintained a continuous curling of the body.

Table 1 lists the stimulation results of the experiments in the flexible plastic film tube. After the above experiments, we summarize the 12 mud eels’ reaction to stimulation as follows. (a) When stimulated, the eels would twist their bodies to try to avoid the stimulation. (b) When stimulated in the trunk, the eels would both twist and curl. The reason for the curling motion was perhaps an attempt to avoid the stimulation: as the eels tried to evade stimulation from the electrodes, they kept bumping the stimulated area against the plastic film tube. (c) When stimulated in the tail, the eels moved forward with relatively large displacement. (d) With increasing distance between the stimulation points, the reaction intensity likewise increased. (e) When simultaneously stimulated in the trunk and tail, the eels would both twist and curl with relatively small forward displacement. Thus, among the various modes of stimulation in the plastic film tube, stimulation in the tail produced the best forward-motion effect.

**Table 1:** **Stimulation results of the experiments in the flexible plastic film tube**

**Stimulating point**	** Reaction**
AS of trunk	Forward and curling motion
MS of trunk	Forward and curling motion
PS of trunk	Curling motion
Tail	Forward motion
AS and MS of trunk	Forward motion
AS and PS of trunk	Curling motion
AS of trunk and tail	Forward and curling motion
MS and PS of trunk	Curling motion

### Stimulation experiments in the simulated intestinal tract

**Figure 10:**
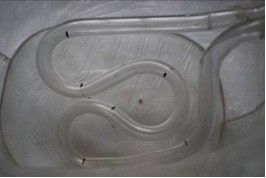
Photo of the simulated intestinal tract.

Since the intestinal tract is long, narrow, and uneven, the intestinal D&T device needs to be flexible. The mean diameter of an unfolded or non-stretched intestinal tract is 20–50 mm; thus, the range of motion for the organism was relatively small. Additionally, the intestinal tract has ascending and descending segments as well as many convoluted segments; therefore, the organism needs to accomplish ascending, descending, and turning motions. The length of the simulated intestinal tract was 1000 mm. It began with a horizontal segment followed by a 180° arc segment, a 360° arc segment, and finally an ascending segment that was 60° to the horizontal plane. Figure 10 shows the simulated intestinal tract. Since the mud eels would be covered with the shell when inside the intestinal tract, the diameter for the range of motion would be less than 20 mm; thus, 15 mm was chosen as the diameter of the simulated tract.

The simulated intestinal tract was narrower than the plastic film tube, and so the eels were unable to curl their bodies, but they were able to move forward, backward, and stop. We divided another 24 mud eels into eight subgroups with three animals in each. Stimulation in the tail was achieved using implanted stimulating electrodes; stimulation elsewhere was done using the wearable stimulating electrodes. As with the experiments in the plastic film tube, we conducted single-point and double-point experiments in the simulated intestinal tract.

**Figure 11:**
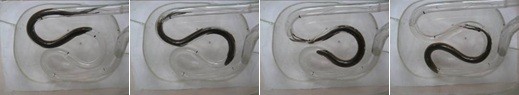
Forward motion when stimulating the tail.

During the experiments, we observed the following motion patterns. (a) When stimulated in the AS, only one of three mud eels moved backward and it did so twice; otherwise, the motion of the three eels was all forward. (b) When stimulated in the MS, all three eels moved forward. (c) When stimulated in the PS, reactions among the three eels varied: one mud eel moved backward three times; the second one moved forward and backward alternately once, but it moved forward twice; the third one moved forward three times. (d) When stimulated in the AS and MS, all three eels moved forward. (e) When stimulated in the AS and PS, two of the three eels moved backward; the other moved forward. (f) When stimulated in the MS and PS, only one of the three eels moved backward and it did so once; the other motion of the three eels was forward. (g) When stimulated in the tail with the implanted stimulating electrodes, all three eels moved forward (Figure 11). (h) When stimulated in the AS and the tail, all three eels moved forward.

Table 2 lists the results of the stimulation experiments in the simulated intestinal tract. After the above experiments, we summarize the 12 mud eels’ reactions to stimulation as follows. (a) When stimulated in the MS, AS and MS, tail, and AS and tail, the eels moved forward. Among those areas, the eels moved fastest when stimulated in the MS of the trunk, which could be used for further studies. Table 3 presents the statistical results of the forward-moving rate in the simulated intestinal tract. (b) When stimulated in the AS, PS, AS and PS, and MS and PS, the eels moved both forward and backward. The reasons for the backward motion may be that (1) the eels attempted to avoid the stimulation by the backward motion or (2) they attempted to avoid the simulated intestinal tract by their backward motion. Elucidating the reason for the backward motion demands further research.

**Table 2:** **Stimulation results of the experiments in the simulated intestinal tract**

**Stimulating point**	** Reaction**
AS of trunk	Forward and backward motion
MS of trunk	Forward motion
PS of trunk	Forward and backward motion
Tail	Forward motion
AS and MS of trunk	Forward motion
AS and PS of trunk	Forward and backward motion
AS of trunk and tail	Forward motion
MS and PS of trunk	Forward and backward motion

**Table 3:** **Statistic results of the forward-moving rate in the simulated intestinal tract**

**Stimulating point**	**Proportion of forward motion**	**Mean forward-moving rate (mm/s)**		
		**Horizontal segment**	**Arc segment**	**Ascending segment**
AS of trunk	77.78%	19.23	18.41	11.47
MS of trunk	100%	21.84	19.12	11.25
PS of trunk	55.56%	/	/	/
Tail	100%	38.64	30.17	20.54
AS and MS of trunk	100%	20.37	20.05	13.09
AS and PS of trunk	33.33%	/	/	/
MS and PS of trunk	88.89%	20.61	18.98	12.54
AS of trunk and tail	100%	22.47	21.04	15.23
Mean rate±standard deviation of each segment (mm/s)		23.86±7.330	21.30±4.445	14.02±3.499
Mean rate±standard deviation of the entire simulative intestinal tract (mm/s)		19.73±6.604		

### Stimulation experiments in the in vitro intestine

We divided another 12 mud eels into two subgroups with six animals in each. One subgroup was used to measure the forward-moving rate in the straight in vitro intestine; the other was used to measure the forward-moving rate in the in vitro intestine with an 80° arc. This experiment was carried out without the shell. We infused clear water into a fresh large intestine of a pig (length, 1000 mm) according to a particular mass ratio (water:mud eel=1:1) for each length of the eel; we did this to simulate the motion in the intestine when the eel was enclosed by the shell. With implantable stimulating electrodes in the middle of the tail, the eels were guided into the intestine. Each stimulation lasted 60 s and was repeated three times.

**Figure 12:**
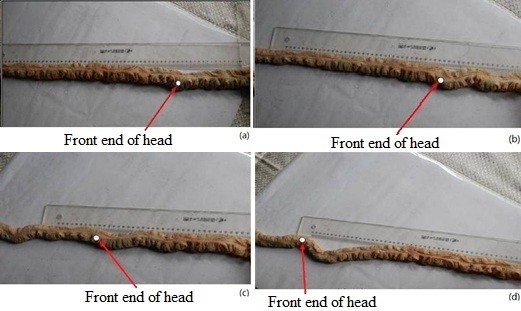
Forward motion when stimulating in the straight intestine.

**Figure 13:**
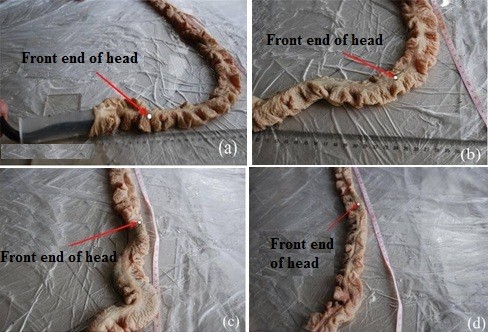
Forward motion when stimulating in the 80° arc intestine.

When stimulated in the straight intestine, the eels clearly moved toward the other end of the intestine during the 60-s stimulation (Figure 12). This result was repeated when they were stimulated in the intestine with an 80° arc (Figure 13). Table 4 lists the forward-moving rate measured in the in vitro intestine.

**Table 4:** Forward-moving rate measured in the in vitro intestine

**Condition of the in vitro intestine**	**Tested mud eel**	**Forward-moving rate (mm/s)**			
		**1**	**2**	**3**	**Mean±Standard deviation**
Straight	A1	18.19	17.5	13.42	17.61±4.3882
	B1	19.07	19.34	10.16	
	C1	32.00	18.05	12.62	
	D1	17.92	17.25	15.80	
	E1	18.64	16.22	15.61	
	F1	19.43	19.06	16.74	
80° arc	A2	16.50	12.52	12.25	15.08±2.0993
	B2	17.05	17.14	16.80	
	C2	16.90	17.26	13.70	
	D2	15.55	15.02	13.87	
	E2	18.10	16.89	12.11	
	F2	14.03	14.10	11.57	

The experimental results with the in vitro intestine indicate that the mud eels were able to move forward over a relatively long distance with 60-s stimulation. The success rate with forward-motion control was over 95%. However, owing to the torsion of the eels, the position of the intestine shifted. The shift was only slight when the eels moved in the straight intestine, though it was severe when they were in the 80° arc intestine. Thus, if the eels were enclosed within a shell, this would weaken the animals’ ability to move within the intestine walls. Further studies are required to eliminate the effect of positional shift of the intestine.

## Conclusions

This study examined an intestinal bio-robot system, which was a combination of organism, neural control, and electromechanical system. The intestinal D&T device was driven by the stimulated organism, and it could actively enter deep parts of the intestine to perform drug release. Using the extremely high energy efficiency of the organism overcomes the problem of power supply to smart capsules. After analyzing the environment in the intestine, we selected the mud eel as the experimental organism: it was appropriate in terms of intestinal conditions and organism controllability. Accordingly, we designed neural microelectrodes and an exterior control unit based on LabVIEW. Through the in vitro stimulation experiments, we derived a method of controlling the organism’s forward motion. Since this is a preliminary study, much work remains to be done, such as improving the design of the shell and establishing an effective method of backward-motion control. However, these results could provide a solid theoretical basis for further research toward designing a practical intestinal bio-robot for diagnosis and treatment of the GI tract.

## Competing interests

The authors declare that they have no competing interests.

## Authors' contributions

LZ and ZYW participated in study design, experiments, data analysis and manuscript preparation. HYL and XTP participated in study design and manuscript preparation. SSZ participated in experiments. All authors read and approved of the final manuscript.
